# Test Performance and Clinical Utility of the Cobas Eplex Blood Culture Identification Fungal Pathogen Panel

**DOI:** 10.1093/ofid/ofag198

**Published:** 2026-04-13

**Authors:** Ashleigh N Riegler, Stefania Carmona, Jeremy Meeder, Derek Moates, Rachael A Lee, Todd P McCarty, Sixto M Leal

**Affiliations:** Department of Pathology, Division of Laboratory Medicine, The University of Alabama at Birmingham, Birmingham, Alabama, USA; Department of Medicine, Division of Infectious Diseases, The University of Alabama at Birmingham, Birmingham, Alabama, USA; Department of Pathology, Division of Laboratory Medicine, The University of Alabama at Birmingham, Birmingham, Alabama, USA; Department of Pathology, Division of Laboratory Medicine, The University of Alabama at Birmingham, Birmingham, Alabama, USA; Department of Medicine, Division of Infectious Diseases, The University of Alabama at Birmingham, Birmingham, Alabama, USA; Department of Medicine, Division of Infectious Diseases, The University of Alabama at Birmingham, Birmingham, Alabama, USA; Department of Pathology, Division of Laboratory Medicine, The University of Alabama at Birmingham, Birmingham, Alabama, USA

**Keywords:** candidemia, fungemia, molecular diagnostics

## Abstract

Identifying fungi to the genus or species level provides valuable insight for guiding antifungal therapy. We evaluated the performance and clinical utility of the Roche cobas eplex blood culture identification fungal pathogen panel (BCID-FP). Although used in fewer than 1% of patients with blood cultures drawn, BCID-FP identified fungal pathogens in more than 96% of tested samples within 72 hours. Over a 3-year period, BCID-FP demonstrated a positive percent agreement (PPA) of 91.7% compared to culture (n = 327). PPA was 100% for *Candida auris*, *Candida kefyr*, *Candida krusei*, *Candida tropicalis*, *Candida parapsilosis*, and *Candida neoformans*; slightly lower for *Candida albicans* (90.3%) and *Candida glabrata* (91.1%); and lowest for *Candida lusitaniae* (55.6%) and *Fusarium* spp. (0%). To assess clinical impact, we compared patient records from preimplementation (results withheld from providers; n = 31) and postimplementation (results released; n = 68). Compared to the T2Dx *Candida* panel, BCID-FP showed superior accuracy (PPA 98.0% vs 61.7%). It also reduced time to fungal identification by 1.36 days relative to culture (*P* < .0001). Postimplementation, there was also a significant increase in infectious disease physician recommendations for antifungal deescalation (39.7% vs 16.1%; *P* = .0219), typically shifting from micafungin to fluconazole in *C. albicans* and *C. parapsilosis* cases. However, no significant differences were observed in time to antifungal optimization, empiric therapy duration, length of stay, or mortality. Although BCID-FP offers clear diagnostic advantages, timely implementation of expert recommendations remains essential to improving outcomes.

Identifying fungi to the genus or species level is critical for guiding antifungal therapy, enabling both deescalation (eg, micafungin to fluconazole) and escalation (eg, micafungin to amphotericin B) [1]. *Candida albicans* remains broadly susceptible to all major classes, with low fluconazole resistance, supporting confident deescalation [2, 3]. Conversely, *C. auris* is frequently multidrug-resistant, requiring susceptibility testing and triggering urgent infection prevention measures [3, 4]. *Candida glabrata* demonstrates elevated minimum inhibitory concentrations to triazoles, making echinocandins the preferred therapy [3]. *Candida parapsilosis* shows higher minimum inhibitory concentrations to echinocandins but is usually fluconazole- and voriconazole-susceptible [3]. *Candida krusei* is intrinsically fluconazole-resistant but treatable with echinocandins or voriconazole [3]. *Candida tropicalis* and *C. kefyr* generally respond to echinocandins, whereas *C. dubliniensis* is fluconazole-susceptible [3]. Less common *Candida. famata* and *Candida. guilliermondii* may exhibit resistance; echinocandins or voriconazole are preferred [3]. *Candida lusitaniae* resists amphotericin B and is best managed with echinocandins [3].

Basidiomycete yeasts, including *Cryptococcus* spp. and *Rhodotorula* spp., are intrinsically echinocandin-resistant [5, 6]. For *Cryptococcus neoformans* and *Candida. gattii*, standard therapy is liposomal amphotericin B plus flucytosine followed by fluconazole [5]. *Rhodotorula* spp. resist triazoles and echinocandins, requiring amphotericin B ± flucytosine [6]. Molds such as *Fusarium* spp. often need voriconazole or lipid amphotericin B, frequently in combination [7]. Early recognition of these pathogens supports rapid therapy optimization and source identification.

These examples underscore the clinical utility of rapid molecular blood culture identification (BCID) panels, which accelerate fungal identification and inform therapy. Despite this promise, few studies have assessed the overall impact of BCID systems on patient outcomes [1, 8, 9]. Importantly, none has focused exclusively on fungi. Prior studies included ≤ 15 fungemia cases, likely because of the increased overall incidence of bacteremia, with conclusions representative of bacteria, not fungi [8, 9].

Currently, 2 commercial polymerase chain reaction–based BCID platforms detect fungi directly from positive blood cultures: bioMérieux BioFire FilmArray BCID2 and the Roche cobas eplex BCID-FP [1]. The latter identifies 15 fungal pathogens, including *C. albicans*, *C. auris*, *C. dubliniensis*, *C. famata*, *C. glabrata*, *C. guilliermondii*, *C. kefyr*, *C. krusei*, *C. lusitaniae*, *C. parapsilosis*, *C. tropicalis*, *C. gattii*, *C. neoformans*, *Fusarium*, and *Rhodotorula*.

Here, we report 3 years of BCID-FP performance data from a major academic medical center, encompassing 331 cases of culture-proven fungemia, with comparison to the T2Dx Candida panel. We further evaluate key clinical outcomes in patients before (results withheld, n = 31) versus after BCID-FP implementation (results reported, n = 68), including time to identification, empiric therapy duration, antifungal optimization, length of stay, and mortality. This study represents the largest fungemia-focused evaluation of a commercial BCID platform to date, addressing a critical gap in the literature.

## MATERIALS AND METHODS

### Study Design

This study was approved by the Institutional Review Board at the University of Alabama at Birmingham (UAB; IRB-300003729). Data were extracted from the UAB Health System Oracle Health electronic medical record (EMR) for patients admitted between January 2020 and December 2024. EMR data from 1 January 2024 to 31 December 2024 was evaluated to characterize blood culture and BCID-FP testing practices (Table 1). EMR data from 2021, 2022, and 2024 (2023 data not available) were evaluated to determine 3-year real-world BCID-FP performance relative to culture (Table 2). To evaluate clinical utility, chart reviews were conducted for patients with culture-confirmed fungemia prior to implementing BCID-FP for patient care (ie, preimplementation phase; May 2020–February 2021; n = 30) and the postimplementation (March 2021–January 2022; n = 70); total n = 105. During preimplementation, BCID-FP was run but results were withheld from providers. In the postimplementation phase, results were reported in real time to guide care. Table 3 summarizes demographics and comorbidities, whereas Table 4 highlights BCID-FP performance with discordant results adjudicated by molecular sequencing. T2Candida Dx panel performance (n = 50) is presented in Table 5. Clinical outcomes across both phases are listed in Table 6.

**Table 1.** Overview of BCID-FP Clinical Testing at UAB Hospital

Category	Total	%
Annual no. of processed blood culture bottles (2024)	53 215	…
**Total no. of processed BC bottles from patient samples** ^a^	52 688	99.0%
**Total no. of patients with blood cultures drawn**	27 094	…
No. with 2 BC bottles (1 set) submitted	25 593	94.5%
No. with only 1 BC bottle submitted	1501	5.5%
**Total no. of unique patients with BCs drawn**	14 530	…
Patientts with an initial fungemia event diagnosed by BC	137	0.9%
**Patients with initial fungemia event also tested with BCID-FP**	125	91.2%
Unique patients with blood cultures tested with BCID-FP	125	0.9%
Invalid tests^c^	4	3.2%
**Patients infected by a fungus on the BCID Panel** ^b^	120	96.0%
Fungi not detected^d^	15	12.5%
Fungi detected	101	84.2%
**Time to fungal ID from BC collection**	…	…
≤ 48 h	78	77.2%
>48 h ≤ 72 h	19	18.8%
>72 h^e^	4	4.0%

Abbreviations: BC, blood culture; BCID-FP, blood culture identification fungal pathogen panel; ID, infectious disease; UAB, University of Alabama at Birmingham.

^a^This total excludes blood products, quality control, proficiency testing, etc.

^b^Five off-panel fungi were present in this cohort: *Candida rugosa*, 2 *Candida* orthopsilosis, 2 *Candida* metapsilosis.

^c^Fungi in the 4 invalid samples were: 2 *Candida glabrata*, 1 *Candida lusitaniae*, 1 *Candida parapsilosis*.

^d^False negatives included: 6 *Candida albicans*, 4 *Candida glabrata*, 1 *Candida dubliniensis*, 4 *Candida lusitaniae*.

^e^Fungi detected >72 h from blood culture collection: 3 *Candida glabrata* (89 h, 93 h, 109 h); 1 *Candida krusei*.

**Table 2.** BCID-FP Performance During 3 Years of Clinical Testing at UAB Hospital

	Total	%	T+	FN	PPA (%)	95% CI	TN	F+	NPA (%)	95% CI
Total no. of BCID-FP tests	336	…	…	…	…	…	…	…	…	…
No. of invalid tests^a^	5	1.5%	…	…	…	…	…	…	…	…
Fungi not on BCID-FP panel^b^	4	1.2%	…	…	…	…	…	…	…	…
Fungi on BCID-FP Panel	327	97.3%	…	…	…	…	…	…	…	…
Samples with fungi Not detected^c^	25	7.6%	…	…	…	…	…	…	…	…
Samples with fungi detected	302	92.4%	279	25	**91**.**8%**	87.9–94.5%	…	4	…	…
*Candida albicans ^d^*	115	38.1%	104	11	**90**.**3%**	83.3–95.0%	212	0	**100**.**0%**	98.4–100%
*Candida auris*	3	1.0%	3	0	**100**.**0%**	29.2–100%	324	0	**100**.**0%**	98.9–100%
*Candida dublinensis*	12	4.0%	11	1	**91**.**7%**	61.5–99.8%	315	0	**100**.**0%**	98.8–100%
*Candida famata*	0	0.0%	0	0	…	…	325	2	**99**.**4%**	97.8–99.9%
*Candida glabrata ^d^*	81	26.8%	74	7	**91**.**1%**	82.6–96.4%	245	1	**99**.**6%**	97.6–99.9%
*Candida guilliermondii*	0	0.0%	0	0	…	…	327	0	**100**.**0%**	98.9–100%
*Candida kefyr*	2	0.7%	2	0	**100**.**0%**	15.8–100%	325	0	**100**.**0%**	98.9–100%
*Candida krusei*	17	5.6%	17	0	**100**.**0%**	80.5–100%	310	0	**100**.**0%**	98.8–100%
*Candida lusitaniae*	9	3.0%	5	4	**55**.**6%**	21.2–86.3%	318	0	**100**.**0%**	98.9–100%
*Candida parapsilosis*	33	10.9%	33	0	**100**.**0%**	89.4–100%	293	1	**99**.**7%**	98.1–99.9%
*Candida tropicalis*	21	7.0%	20	1	**95**.**2%**	76.2–99.9%	306	0	**100**.**0%**	98.8–100%
*Cryptococcus gatii*	0	0.0%	0	0	…	…	327	0	**100**.**0%**	98.9–100%
*Cryptococcus neoformans*	10	3.3%	10	0	**100**.**0%**	69.2–100%	317	0	**100**.**0%**	98.8–100%
*Fusarium* spp.	1	0.3%	0	1	**0**.**0%**	0.00–97.5%	326	0	**100**.**0%**	98.9–100%
*Rhodotorula* spp.	0	0.0%	0	0	…	…	327	0	**100**.**0%**	98.9–100%

Abbreviations: BCID, blood culture identification panel; CI, confidence interval; FN–, false negative; F+, false positive; FP, fungal panel; NPA, negative percent agreement; PPA, positive percent agreement; TN–, true negative; T+, true positive.

^a^Fungi in the 5 invalid samples were: 3 *Candida glabrata*, 1 *Candida lusitaniae*, 1 *Candida parapsilosis*.

^b^Four off-panel fungi were present in this cohort: 2 *Candida orthopsilosis*, 2 *Candida metapsilosis*.

^c^False negatives included: 11 *Candida albicans*, 7 *Candida glabrata*, 4 *Candida lusitaniae*, 1 *Candida dubliniensis*, 1 *Candida* trop, 1 *Fusarium*.

^d^Two samples exhibited mixed infection with *Candida albicans* and *Candida glabrata*; each species was included in the performance characteristic evaluation, hence total samples with on panel fungi detected in culture = 327 but the total number of on-panel isolates = 329.

95% Confidence Interval calculated by Clopper-Pearson method, (https://www.medcalc.org/calc/diagnostic_test.php).

**Table 3.** Patient Characteristics in the Pre- and Post-BCID-FP Implementation Period

Total Cases	All Study Samples		Pre Implementation		BCID-FP Implementation		
	105		35		70		
**Patient demographics**	**Mean**	**SEM**	**Mean**	**SEM**	**Mean**	**SEM**	***P* Value ^a^**
**Age [y]**	52.2	1.51	56	2.73	50	1.77	.066
	**No.**	**%**	**No.**	**%**	**No.**	**%**	…
**Sex**	…		…		…	…	…
Male	59	56.2%	24	68.6%	35	50.0%	.066
Female	46	43.8%	11	31.4%	35	50.0%	
**Race/ethnicity**	…		…	…	…	…	…
White	60	57.1%	18	51.4%	42	60.0%	.405
Black	39	36.8%	14	40.0%	25	35.2%	.669
Asian	4	3.8%	3	8.6%	1	1.4%	.104
Hispanic/Latino	2	1.9%	0	0.0%	2	2.8%	>.999
**Chronic comorbidities**							
**Immunosuppression**	61	57.5%	24	68.6%	37	52.1%	.144
Solid malignancy	21	19.8%	10	28.6%	11	15.5%	.127
Hematologic malignancy	8	7.5%	2	5.7%	6	8.5%	>.999
Solid organ transplant	12	11.3%	5	14.3%	7	9.9%	.525
Hematopoietic stem cell transplant	2	1.9%	0	0.0%	2	2.8%	>.999
Other (steroids, biologics, etc.)	18	17.0%	7	20.0%	11	15.5%	.589
**Diabetes**	27	25.5%	11	31.4%	16	22.5%	.350
**Cardiovascular disease**	13	12.3%	6	17.1%	7	9.9%	.348
**Chronic lung disease**	15	14.2%	3	8.6%	12	16.9%	.376
**Chronic kidney disease**	10	9.4%	4	11.4%	6	8.5%	.727
**Cirrhosis**	5	4.7%	2	5.7%	3	4.2%	>.999
**Human immunodeficiency virus Positive**	3	2.8%	2	5.7%	1	1.4%	.*668*
**Intervention or diagnosis**							
**Mechanical ventilation**	52	49.1%	16	45.7%	36	50.7%	.*683*
**ECMO**	13	12.3%	1	2.9%	12	16.9%	.*056*
**Trauma at the time of admission**	2	1.9%	1	2.9%	1	1.4%	*>*.*999*
…	**Mean**	**SEM**	**Mean**	**SEM**	**Mean**	**SEM**	…
**Pitt Bacteremia Score**	4.01	0.332	4.11	0.521	3.96	0.426	.*826*

Abbreviations: BCID-FP, blood culture identification fungal pathogen panel; ECMO, extracorporeal membrane oxygenation; SEM, standard error of the mean.

^a^Statistical significance for continuous data was assessed using unpaired *t*-tests, wheres categorical data was analyzed with Fisher's exact test; Comparisons are between preimplementation and BCID-FP implementation.

**Table 4.** BCID-FP Performance During the Pre- and Post-BCID-FP Implementation Period

	Total	%	T+	FN	PPA (%)	95% CI	TN	F+	NPA (%)	95% CI
Total no. BCID-FP tests	105	…	…	…	…	…	…	…	…	…
Invalid test^a^	3	2.9%	…	…	…	…	…	…	…	…
Fungi NOT on BCID Panel^b^	3	2.9%	…	…	…	…	…	…	…	…
Fungi on BCID-FP Panel	99	94.3%	…	…	**…**	…	…	…	**…**	…
Samples with fungi not detected^c^	2	2.0%	…	…	…	…	…	…	…	…
Samples with fungi detected	97	98.0%	97	2	**98**.**0%**	92.9–99.8%	…	1	…	…
*Candida albicans ^d^*	50	51.5%	49	1	**98**.**0%**	89.3–99.9%	48	1	**98**.**0%**	89.2–99.9%
*Candida dublinensis*	4	4.1%	4	0	**100**.**0%**	39.8–100%	95	0	**100**.**0%**	96.2–100%
*Candida glabrata ^d^*	17	17.5%	17	0	**100**.**0%**	80.5–100%	82	0	**100**.**0%**	95.6–100%
*Candida kefyr*	1	1.0%	1	0	**100**.**0%**	2.5–100%	98	0	**100**.**0%**	96.3–100%
*Candida krusei*	6	6.2%	6	0	**100**.**0%**	54.1–100%	93	0	**100**.**0%**	96.1–100%
*Candida lusitaniae*	1	1.0%	0	1	**0**.**0%**	0–97.5%	98	0	**100**.**0%**	96.3–100%
*Candida parapsilosis*	13	13.4%	13	0	**100**.**0%**	75.3–100%	86	0	**100**.**0%**	95.8–100%
*Candida tropicalis*	7	7.2%	7	0	**100**.**0%**	59.0–100%	92	0	**100**.**0%**	96.1–100%
*Cryptococcus neoformans*	2	2.1%	2	0	**100**.**0%**	15.8–100%	97	0	**100**.**0%**	96.3–100%

Abbreviations: BCID-FP, blood culture identification fungal panel; CI, confidence interval; F+, false positive; FN, false negative; NPA, negative percent agreement; PPA, positive percent agreement; TN, true negative; T+, true positive.

^a^Organisms in the 3 invalid samples were: 2 *Candida albicans*, 1 *Candida lusitaniae*.

^b^Three fungi not on the BCID FP panel were: 1 *Candida bracariensis*, 1 *Candida metapsilosis*, and 1 *Candida pararugosa*.

^c^False negatives included: 1 *Candida albicans* in a mixed infection with *Candida glabrata*; 1 *Candida lusitaniae* called *Candida albicans*.

^d^Two samples exhibited mixed infection with *Candida albicans* and *Candida glabrata*; each species was included in the performance characteristic evaluation, hence total samples with on panel fungi detected in culture = 99 but the total #number of on-panel isolates = 101.

95% Confidence Interval calculated by Clopper-Pearson method, (https://www.medcalc.org/calc/diagnostic_test.php)

**Table 5.** T2CandidaDx Performance During the Pre- and Post-BCID-FP Implementation Period

	Total	%	T+	FN	PPA (%)	95% CI	TN	F+^d^	NPA (%)	95% CI
Total no. T2 tests^a^	50	…	…	…	…	…	…	…	…	…
Fungi NOT on T2 panel^b^	3	6.0%	…	…	…	…	…	…	…	…
Fungi on T2 panel	47	94.0%	29	18	**61**.**7%**	46.4–75.5%	…	10	**…**	…
*Candida albicans (A/T) ^c^*	26	55.3%	16	10	**61**.**5%**	40.6–79.8%	17	4	**81**.**0%**	58.1–94.6%
*Candida tropicalis (A/T)*	3	6.4%	2	1	**66**.**7%**	4.3–77.7%	40	4	**90**.**9%**	87.1–99.9%
*Candida krusei (K/G)*	4	8.5%	2	2	**50**.**0%**	6.8–93.2%	42	1	**97**.**7%**	87.8–99.9%
*Candida glabrata (K/G) ^c^*	9	19.1%	5	4	**55**.**6%**	21.2–86.3%	37	1	**97**.**4%**	86.2–99.9%
*Candida parapsilosis (P)*	5	10.6%	4	1	**80**.**0%**	28.4–99.5%	42	0	**100**.**0%**	91.6–99.9%

Abbreviations: CI, confidence interval; F+, false positive; FN, false negative; FP, fungal panel; NPA, negative percent agreement; PPA, positive percent agreement; T+, true positive; TN, true negative.

^a^Samples evaluated by BCID FP, T2CandidaDx, and culture.

^b^Three fungi not on the T2 panel were: 1 *Candida lusitaniae*, 1 *Candida bracariensis*, 1 *Candida neoformans*.

^c^One mixed infection had both *Candida albicans* and *Candida glabrata*.

^d^False positives included A/T results on 1 *Candida krusei*, 2 C. *glabrata*, 1 *Candida parapsilosis*, and a K/G result on 1 *Candida bracariensis*. Because A/T or K/G results yield false positives for 2 organisms, the total T2 panel false-positive rate takes into consideration 4 false positives (not 8) in this cohort of samples.

95% Confidence Interval calculated by Clopper-Pearson method, (https://www.medcalc.org/calc/diagnostic_test.php).

**Table 6.** Clinical Utility of the BCID-FP

	Total		Preimplementation		BCID-FP Implementation		*P Value ^e^*
Patients witd fungemia identified by culture	105		35		70		
No. invalid BCID-FP tests^a^	3		3		0		
No. off-panel fungi^b^	3		1		2		
Fungi Identified by BCID-FP	99		31		68		
	**Mean**	**SEM**	**Mean**	**SEM**	**Mean**	**SEM**	
Time from BC collection to culture ID (days)^c^	3.14	0.11	3.18	0.19	3.12	0.13	.7824
**Time from BC collection to BCID-FP ID^c^**	**1.78**	**0.07**	**2.07**	**0**.**17**	**1.65**	**0**.**07**	.**0071**
***t*-test on BCID-FP time to ID versus culture**							**<.0001**
Mean time BCID-FP saved to fungal ID^d^	1.36	0.09	1.12	0.09	1.47	0.12	.0727
	***No.***	***%***	***No.***	***%***	***No.***	***%***	
Empiric therapy before culture collection	10	10.1%	2	5.7%	8	8.1%	.4992
…	**Mean**	**SEM**	**Mean**	**SEM**	**Mean**	**SEM**	
Time on empiric therapy	1.18	0.45	1.83	1.65	0.91	0.41	.*5003*
Associated toxicity	None Identified						
	**No.**	**%**	**No.**	**%**	**No.**	**%**	
**Expert recommended deescalation^f^**	**32**	**32.3%**	**5**	**16**.**1%**	**27**	**39**.**7%**	.**0219**
Deescalation implemented^g^	22	22.2%	5	16.1%	17	25.0%	.4368
	**Mean**	**SEM**	**Mean**	**SEM**	**Mean**	**SEM**	
Time from BC collection to deescalation	2.48	0.44	2.60	0.40	2.45	0.60	.8958
	**No.**	**%**	**No.**	**%**	**No.**	**%**	
Expert recommended escalation^f^	2	2.0%	1	3.2%	1	1.5%	.5304
Escalation implemented^h^	2	2.0%	1	3.2%%	1	1.5%%	.5304
	**Mean**	**SEM**	**Mean**	**SEM**	**Mean**	**SEM**	
Time from BC collection to escalation	0.89	0.11	1.00	N/A	0.78	N/A	N/A
LoS	23.3	2.7	16.9	3.4	26.1	3.8	.1149
LoS estimated cost per patient^i^	$70 391.51		$47 160.00		$79 118.00		
LoS: ECMO patients excluded	19.5	2.3	17.5	3.4	20.4	2.9	.5480
LoS estimated cost/patient; ECMO excluded^i^	$58 987.50		$52 937.50		$61 710.00		
	***No.***	***%***	***No.***	***%***	***No.***	***%***	
7-day mortality	25	25.3%	8	25.8%	17	25.0%	>.9999
30-d mortality	50	50.5%	17	54.8%	33	48.5%	.6658

Abbreviations: BCID-FP, blood culture identification-fungal panel; CI, confidence interval; Dz-disease; F+, false positive; FN, false negative; ID, identification; LoS, length of stay; NPA, negative percent agreement; PPA, positive percent agreement; SEM, standard error of the mean; Sn, sensitivity; Sp, specificity; T+, true positive; TN, true negative; Tx, treatment.

^a^Organisms in the 3 invalid samples were: 2 *Candida* albicans, 1 *Candida lusitaniae*.

^b^Three fungi not on the BCID FP panel were: 1 *Candida bracariensis*, 1 *Candida metapsilosis*, and 1 *Candida pararugosa*.

^c^Time to ID calculated from initial blood draw.

^d^Time saved to organism ID compared to culture standard of care.

^e^Statistical significance for continuous data was assessed using unpaired *t*-tests, while categorical data was analyzed with Fisher's exact test; Unless otherwise indicated *P* values correspond to comparisons between pre- and postimplementation cohorts.

^f^Escalation or deescalation recommended by expert infectious disease physicians upon chart review.

^g^All 22 deescalations involved a switch from micafungin to fluconazole; preimplementation: 3 *Candida albicans*, 2 *Candida parapsilosis*; postimplementation: 13 *Candida albicans*, 1 *Candida dubliniensis*, 4 *Candida parapsilosis* (1 mixed with *Candida albicans*).

^h^Escalation in the preimplementation phase involved a switch from micafungin to amphotericin B and flucytosine to treat 1 *Candida* neoformans. Escalation in the postimplementation phase involved a switch from fluconazole to micafungin to treat 1 *Candida albicans*.

^i^Length of stay estimated cost per patient determined by multiplying the LoS by the US average cost of inpatient hospitalization ($3025) as reported by the North American Community Hub Statistics—NCHStats.

Metrics included: time to organism identification by culture and BCID-FP; mean identification time saved; empiric antifungal duration and toxicity; time to expert-recommended therapy changes; adherence to those recommendations; length of stay (LoS); per-patient cost estimates (with and without extracorporeal membrane oxygenation [ECMO]); and 7-day and 30-day mortality. LoS costs were estimated by multiplying hospital days from culture collection to discharge by the 2025 U.S. average inpatient cost ($3025/day; North American Community Hub Statistics). Estimated LoS is provided for patients that received or did not receive ECMO but does not incorporate the specific cost associated with this procedure.

### Sample Collection and Processing

The standard diagnostic pathway for bloodstream infection at UAB remained unchanged across study phases, aside from BCID-FP reporting. Peripheral blood was collected in bioMérieux BacT/Alert resin FA/FN bottles and incubated in the BacT/Alert VIRTUO system. Positive bottles underwent gram stain and subculture onto solid media. Isolates were identified by bioMérieux VITEK MS MALDI-TOF MS; molds were further characterized by tape prep microscopy. Gram stains positive for fungal elements triggered BCID-FP testing of a blood culture aliquot. In select cases (n = 50), providers additionally ordered T2Candida Dx testing on ethylenediaminetetraacetic-anticoagulated blood at the time of blood culture collection. This synchronous collection enabled the direct comparison of test performance (PPA/NPA) for the identification of yeasts on both molecular panels. Note that time to identification was not compared, given inherent differences in directly evaluating blood versus a positive blood culture bottle.

### Statistical Methods

Positive percent agreement and NPA were calculated by standard methods. Two-sided 95% confidence intervals were estimated using the Clopper–Pearson method. Continuous variables were analyzed using unpaired *t*-tests, whereas categorical comparisons used Fisher's exact test. Analyses were performed in Prism (GraphPad Software, Boston, MA), with *P* < .05 considered statistically significant.

## RESULTS

### BCID-FP Identified the Causative Organism in 96% of Cases Within 72 hours of Collection

In 2024, 53,215 blood culture bottles were processed at UAB Hospital, with 52 688 (99.0%) from patient samples (Table 1). Cultures were drawn from 27 094 patients; 25 593 (94.5%) had 2 bottles submitted and 1501 (5.5%) had 1 bottle. Among 14 530 unique patients, 137 (0.9%) had fungemia by culture; 125 (91.2%) of these underwent BCID-FP based on yeast seen on gram stain. Four BCID-FP tests (3.2%) were invalid (2 *C. glabrata*, 1 *C. lusitaniae*, 1 *C. parapsilosis*). Five patients (4.0%) with fungemia were infected with fungi not present on the BCID-FP panel (*Candida rugosa*; 2 *Candida orthopsilosis*; 2 *Candida metapsilosis*). Fifteen cases (12.5%) were BCID-FP negative despite yeast on gram stain (6 *C. albicans*, 4 *C. glabrata*, 1 *C. dubliniensis*, 4 *C. lusitaniae*). Overall, fungi were detected in 101/120 interpretable cases (84.2%). From blood culture collection, 77.2% of BCID-FP results were available in <48 hours, 18.8% at 48–72 hours, and 4.0% > 72 hours, including 3 *C. glabrata* detections at 89, 93, and 109 hours and 1 *C. krusei* at 97 hours.

### BCID-FP Demonstrated High Concordance With Blood Culture Over a 3-year Period

Across 336 BCID-FP tests, 5 (1.5%) were invalid and 4 (1.2%) targeted off-panel fungi (2 *C. orthopsilosis*, 2 *C. metapsilosis*). Of 327 valid, on-panel tests, 302 (92.4%) were positive and 25 (7.6%) negative (Table 2). Overall agreement with culture was high: PPA 91.7% (true positive 275, false negative 25). False negatives comprised 11 *C. albicans*, 7 *C. glabrata*, 4 *C. lusitaniae*, 1 *C. dubliniensis*, 1 *C. tropicalis*, and 1 *Fus*. The PPA and NPA for each species are outlined in Table 2.

### Patient Demographics and Comorbidities Were Comparable Pre- and Post-BCID-FP Implementation

We enrolled 105 unique patients—35 preimplementation (BCID-FP results withheld from providers) and 71 postimplementation (results reported). Mean age was 52.2 years (standard error of the mean 1.51); preimplementation patients were slightly older (56 vs 50 years) (Table 3). Males comprised 55.7% overall (68.6% pre vs 49.3% post). Racial/ethnic distribution was 59.4% White, 36.8% Black, 3.8% Asian, and 1.9% Hispanic/Latino, without significant between-group differences. Immunosuppression was common (57.5% overall; 68.6% pre vs 52.1% post) and included solid malignancy (19.8%), hematologic malignancy (7.5%), solid organ transplant (11.3%), hematopoietic stem-cell transplant (1.9%), and steroid/biologic use (17.0%). Other comorbidities included diabetes (25.5%), cardiovascular disease (12.3%), chronic lung disease (14.2%), chronic kidney disease (9.4%), cirrhosis (4.7%), and human immunodeficiency virus (2.8%). Nearly half required mechanical ventilation (49.1%; 45.7% pre vs 50.7% post). ECMO was used in 12.3% overall (16.9% post vs 2.9% pre). Trauma at admission was rare (1.9%). Mean Pitt Bacteremia Score was 4.01 (standard error of the mean 0.332) without group differences. Statistical tests used unpaired *t*-tests (continuous) and Fisher's exact (categorical).

### BCID-FP Demonstrated High Concordance With Culture Pre- and Post-BCID Implementation

Of 105 trial samples, 3 (2.9%) were invalid (2 *C. albicans*, 1 *C. lusitaniae*), and 3 (2.9%) were off-panel (*Candida bracarensis*, *C. metapsilosis*, *C. parapsilosis*) (Table 4). Among 99 valid, on-panel tests, 97 were true positives with 2 false negatives (PPA 98.0%). Fungi were detected in 97/99 valid tests (98.0%). *C. albicans* was most common (50/97; 51.5%) and had 1 false negative (FN) and 1 false positive (FP) (PPA 98.0, NPA 98.0). *C. glabrata* (17), *C. dubliniensis* (4), *C. kefyr* (1), *C. krusei* (6), *C. parapsilosis* (13), *C. tropicalis* (7), and *C. neoformans* (2) each showed 100% agreement. *C. lusitaniae* produced a single FN via misidentification as *C. albicans* (species-level PPA 0.0, NPA 100.0). The two FNs were: (1) mixed *C. albicans/C. glabrata* and (2) *C. lusitaniae* called as *C. albicans*. Two samples had mixed *C. albicans/C. glabrata* infections.

### T2Candida Dx Exhibited Poor Performance Pre- and Post-BCID Implementation

Fifty samples were tested in parallel by T2Candida, BCID-FP, and culture (**Table 5)**. Three (6.0%) contained off-panel fungi for T2 (*C. lusitaniae*, *C. bracarensis*, *C. neoformans*), leaving 47 on-panel. Overall, T2 showed PPA 61.7% (29 TP, 18 FN) and NPA 78.7% (37 TN, 10 FP). The PPA and NPA for each species is outlined in Table 5. One mixed infection included *C. albicans/C. glabrata*. False positives occurred with *C. krusei*, *C. glabrata*, and *C. parapsilosis*, often driven by cross-reactive A/T or K/G group signals; because T2 reports grouped targets, overlapping calls translated to 4 unique FP events. These data demonstrate substantially lower sensitivity and specificity for T2 relative to BCID-FP in this cohort.

### BCID-FP Improved the Time to Identification and Rate of Expert Recommended Antifungal de-escalation

Among 105 fungemic patients (35 preimplementation, 70 postimplementation), 3 BCID-FP tests were invalid (2 *C. albicans*, 1 *C. lusitaniae*; all preimplementation), and 3 were off-panel (*C .bracarensis*, *C.metapsilosis*, *C. parapsilosis*) (Table 6). BCID-FP identified fungi in 99 cases (31 preimplementation; 68 postimplementation). Mean time from blood-culture collection to fungal identification by culture was 3.14 days with no pre/post difference (3.18 vs 3.12 days). BCID-FP reduced time to identification to 1.78 days, overall, faster postimplementation (1.65 vs 2.07 days), yielding a mean time saved of 1.36 days relative to culture.

Empiric antifungal therapy before culture collection was uncommon (10.1% pre vs 11.4% post), with short duration (mean 1.18 days) and no identified toxicity. Expert retrospective review by mycology-focused infectious disease (ID) physicians recommended deescalation in 32.3% overall, more often postimplementation (39.7%) than preimplementation (16.1%). This expert review and recommendation was not available during the care of patients enrolled in this study. In contrast, additional chart review was performed to determine if providers taking care of patients in this study in real time chose to deescalate antifungal therapy based on data available to them in the EMR. In this real-world analysis, deescalation occurred in 22.2% of cases overall: all 5 retrospective ID recommendations in the preimplementation phase had been implemented (despite no BCID-FP data), and 17 of 27 (63.0%) ID recommendations in the postimplementation phase were acted on. All deescalations were micafungin → fluconazole. Preimplementation deescalations involved 3 *C. albicans* and 2 *C. parapsilosis*; postimplementation deescalations involved 13 *C. albicans*, 1 *C. dubliniensis*, and 4 *C. parapsilosis*, including 1 mixed *C. albicans* case.

Management changes were recommended in 2.0% of cases (1 case per phase) and implemented in both. Preimplementation, micafungin was escalated to amphotericin B + flucytosine for *C. neoformans*; postimplementation, fluconazole was escalated to micafungin for *C. albicans*. Mean time to changes in therapy was 0.89 days from blood-culture collection.

Length of stay (LoS) did not differ significantly by phase. Overall mean LoS was 23.3 days (16.9 pre vs 26.1 post). Excluding ECMO patients, mean LoS was 19.5 days (17.7 pre vs 20.4 post). Estimated cost per patient was higher postimplementation ($79 118) than preimplementation ($47 160), with an overall mean of $70 391; excluding ECMO, mean cost was $58 987 ($52 938 pre vs $61 710 post). Seven-day mortality was similar (25.3% pre vs 25.8% post). Thirty-day mortality trended lower postimplementation (48.5% vs 54.8%) without statistical significance.

## DISCUSSION

In this study, BCID-FP demonstrated high concordance with culture while reducing the time to species identification by 1.36 days and increasing the rate of ID physician recommendations for antifungal deescalation by 23.6% (39.7% postimplementation minus 16.1% preimplementation). Compared to the Food and Drug Administration–cleared T2Candida panel, BCID-FP showed superior PPA (98.0% vs 61.7%). However, despite these diagnostic advantages, there were no statistically significant differences in time to antifungal optimization, empiric therapy duration, length of stay, or mortality.

The literature on BCID-FP test performance includes three studies with a total of 5, 11, and 15 fresh clinical samples from patients with fungemia [1, 8–10]. In contrast, we evaluated 336 fresh clinical samples tested in a real-world setting over 3 years. Prior to this study, Zhang et al. published the largest BCID-FP evaluation, which included 21 prospective clinical samples (10 frozen, 11 fresh), 120 retrospective frozen samples, and 726 contrived samples [10]. We analyzed the following distribution of prospective fresh samples: *C. albicans* (n = 113), *C. auris* (n = 3), *C. dubliniensis* (n = 12), *C. glabrata* (n = 79), *C. kefyr* (n = 2), *C. krusei* (n = 17), *C. lusitaniae* (n = 9), *C. parapsilosis* (n = 33), *C. tropicalis* (n = 21), *C. neoformans* (n = 10), and *Fusarium fus* (n = 1). *Rhodotorula* was only identified by Zhang et al. (2 cases), and neither study detected *C. famata*, *C. guilliermondii*, or *C. gattii* [10].

Relative to a controlled research environment, we expected worse test performance in the patient care setting; however, we observed an improved validity rate (98.5% vs 96.8%) compared with Zhang et al [10]. This was likely due to 100% fresh clinical samples in our study versus 1.2% (n = 10). Studies by Huang et al. (n = 7; Belgium) and Bryant et al. (n = 15; France) did not report invalid results for fungemia samples [8, 9]. Both studies, however, evaluated all 3 BCID panels in populations dominated by bacteremia, obscuring the specific performance of BCID-FP [8, 9].

In contrast, the BCID-FP PPA (91.7%) in this study was lower than the 97.2% reported by Zhang et al [10]. Species-level PPA values were similar, with both studies identifying *C. lusitaniae* as the lowest performer (55.6% vs 75%) [10]. Although *Fusarium* spp. are the most common molds isolated from standard blood culture bottles, they are associated with a clinical syndrome distinct from yeast fungemia and given the small sample size, no claims are made on this panel's ability to identify this mold. BCID-FP cannot detect all *Fusarium* species and has diminished capacity for some, such as *Fusarium rodolens,* which is not predicted to be detected at the genus level [11]. Without species-level identification, this *Fusarium* strain may represent an off-panel organism and thus a true negative. Discordant analyses likely would have resolved some of the 25 FNs, yielding higher PPA [8, 12]. Notably, during the pre- and postimplementation phases (n = 105) where sequencing was performed, the PPA reached 98.0%.

Compared with bacteria, yeast colonies have less morphological variability, increasing the risk of missing a second yeast in mixed culture. This may explain a subset of FNs and highlights the importance of parallel culture on solid media to complement BCID-FP. Despite these limitations, the panel demonstrated 100% specificity across most targets and accurately identified 2 mixed infections (*C. albicans*/*C. glabrata*), confirming utility in polymicrobial fungemia.

Our data highlight the diagnostic stewardship value of the gram stain, which focused BCID-FP testing to just 0.9% (n = 137) of samples among 27 094 patients with blood cultures in 1 year. BCID-FP accurately detected pathogens in all 10 patients already on empiric antifungals (8 on micafungin, 2 on fluconazole), supporting its reliability in real-world use.

The overall NPA in our study (98.9%) was consistent with prior reports [8–10]. Without discordant analyses in the 3-year dataset, some of the 4 apparent FPs may have been true positives. A similar NPA (99%) was observed in the subset with sequencing, reinforcing high confidence in negative results and supporting exclusion of on-panel fungi when BCID-FP is negative.

By contrast, T2Candida performed poorly, with low sensitivity (61.7%) and specificity (78.7%), a high FN rate (38.3%), and frequent false positives (21.3%). Species-level sensitivity was also suboptimal: *C. albicans* (61.5%), *C. glabrata* (55.6%), *C. krusei* (50%), and *C. parapsilosis* (80%). These findings align with prior reports of variable T2 performance [13–16]. This study is the first direct comparison of T2Candida with a rapid molecular blood culture platform, demonstrating superior BCID-FP performance in sensitivity, specificity, and resolution. Because T2 detects fungal DNA directly from blood regardless of viability, culture may not be the ideal comparator. T2Candida is now discontinued.

Patient demographics and comorbidities were similar pre- and post-BCID-FP, though some trends suggested confounders. Immunosuppression was more frequent preimplementation. ECMO use was higher postimplementation, overlapping with the coronavirus disease 2019 pandemic, which likely influenced intensive care unit access and length of stay [17]. Excluding ECMO patients reduced length-of-stay differences, suggesting illness severity or access to advanced care as confounders. Interpretation of the effect of the assay on cost and length of stay requires caution due to these confounding variables.

BCID-FP reduced mean time to identification from 3.14 to 1.78 days and increased expert recommendations for antifungal deescalation 2.5-fold (39.7% postimplementation divided by 16.1% preimplementation). The most common recommendation was switching from micafungin to fluconazole. Escalation was rare (2%) but facilitated optimized treatment for critical infections with *C. albicans* and *Cryptococcus neoformans*. Notably, recommendations for azole or echinocandins as first-line therapy are implemented inconsistently both nationally and globally [18, 19]. This inconsistency along with geographic shortages in clinicians who specialize in ID and can recognize infectious syndromes before pathogen identification support the implementation of tests, which can provide earlier pathogen identification to aid in treatment selection or warrant transfer to an institution with ID consultation available [20, 21].

Our findings mirror 2 studies that reviewed charts to evaluate BCID impact [8, 9]; however, both included >95% bacteremia. Bryant et al. concluded that 11% of patients might have benefited from BCID-FP data but included only 15 fungemia cases and lacked a control group [8]. In 2024, Caspar et al. conducted a randomized trial comparing BCID testing (n = 105, 3 fungemia) to controls (n = 107, 5 fungemia) [9]. Although shorter times to therapy were observed, benefits likely reflected BCID-GP and GN panels, as 96% of samples were from patients with bacteremia [9]. No other published studies have specifically evaluated BCID-FP clinical utility.

Our dataset (n = 99) is the largest to date assessing BCID-FP clinical utility. Despite more cases, trends such as reduced 30-day mortality postimplementation (54.8% vs 48.5%) were not statistically significant. Larger sample sizes or more standardized study designs may be needed to detect outcome differences.

The Caspar trial design relied on antimicrobial stewardship enrollment, with close monitoring and rapid dissemination of BCID results [9]. At our institution, in the preimplementation phase, a gram stain result prompted ID consult within the same day (before 4 Pm weekdays or 2 Pm weekends) and phone communication within 30 minutes. However, postimplementation, gram stain results were withheld until BCID-FP results were available, producing a single call with both gram stain and identification within 2 hours. This streamlined approach may have delayed consult initiation by approximately 90 minutes, potentially mitigating BCID-FP impact on outcomes. Additionally, although an ID consult was initiated for each case of fungemia, this study did not evaluate the potential confounding variables of time to ID review of consult cases or time to provision of expert recommendations.

Retrospective review of post-implementation cases by expert ID providers identified the need for deescalation in 39.7%. However, in real-time, the providers taking care of these patients only deescalated therapy in 25% of cases, highlighting the gap between diagnostic potential and clinical adoption. Strategies such as real-time EMR alerts, treatment templates, automatic consults to Infectious Diseases/Antimicrobial Stewardship Program, pharmacist involvement, and stewardship team routing may improve integration [22, 23]. However, effectiveness depends on institutional resources and provider engagement. Feedback loops, education, and cultural change are required for sustainable adoption. Translating theoretical benefits into practice remains a work in progress.
